# Healthy Eating

**Published:** 1875-09

**Authors:** 


### HEALTHY EATING.

A good deal of advice is given to the American public, from time to time, as to what constitutes a healthy diet, both as to the dishes of which such a diet should be made up, and as to the manner in which they should be prepared. But, while it is almost hopeless to get any general agreement in regard to these matters, we believe there is one suggestion that could be considered with advantage by every one, whatever his tastes may be as to the food he selects or the way in which he likes it cooked. That suggestion is very simple. It is that all food should be thoroughly chewed before being swallowed. Whether we like hearty meals or light ones; whether we prefer our meats roasted, or broiled, or fried; whether we take kindly to bread and vegetables, or incline to pastry, it is certain that the observance of this simple rule will go a great way toward protecting us from injury, and securing for us the full benefit of what we eat. So nicely, indeed, is the machinery of digestion adjusted, that this single precaution may be relied upon to exclude from our plates, or at least from our palates, any variety of food that is seriously unfitted to nourish the system.

The physiological basis of the rule is very simple, and will not, we think, be disputed by any one. It lies in the fact that the effects, both mechanical and chemical, of thorough mastication, are the same as those secured by the action of the lower digestive organs. We say lower because, as a matter of fact, the teeth and the salivary glands are as essentially, in the intention of nature, a part of the digestive organs, as the stomach and the liver. The service which the saliva is capable of performing, if we would only give it time and opportunity, is strongly analogous to, if not indentical with, that required of the gastric juice. And in a general way, it may be said that the more nearly the food is reduced to fine pulp in the mouth, the less remains for the other portions of the digestive apparatus to do, the more easily and completely their task is performed, and the more perfect is the preparation of the food for its purpose—the formation of blood and the nutrition of the whole body.

These are very elementary truths, but they are little known and less regarded. If we are content to ignore them for ourselves, as is the case with most of even those who are entirely familiar with them, we might, at least, enforce them on the attention of our children. We could hardly do these little creatures a greater service than to require them to form the habit of eating slowly, in small mouthfuls, and masticating their food well. LA FONTAINE says, in the preface to the first edition of his fables, that "if we would not be reduced to correcting our habits we must labor to form good ones in minds that are as yet indifferent to good or ill," and the wise saying of the witty moralist of 200 years ago is confirmed by the latest and most complete physiological research. Two results are sure to flow from a careful habit of eating: one that we will not eat too much, and the other that we will gradually acquire an instinctive aversion to unhealthy food. When a man "bolts" his food, giving it only a momentary opportunity to gratify the palate, and then sending it headlong to the stomach, he is liable to take a great deal more than he can readily digest because the sense of taste, only rapidly and slightly exerting itself, is not sated until long after the amount has been exceeded of which the stomach can properly dispose. And on the other hand, in the rapid process, the taste has no opportunity to assert its real power of choice. It is placed at the entrance of the digestive system, not only as one of the most important members of it, but as a guard and sentinel to protect the other members. But what can be expected of it when we crowd upon and past it a mass of matter which it has no time to examine. Treat it fairly, and the sense of taste would demonstrate its power to do ample justice to its duties, and to this end nothing is necessary but an adequate reduction of the food by the combined working of the teeth and the salivary secretions. And it may be added that adhesion to the very simple rule suggested would not only be of the greatest benefit to health, but would indefinitely increase the pleasures of the table. Those who follow it will steadily develop their capacity to tell good from bad or indifferent food, and will, moreover, increase their natural and delicate enjoyment of the former.—*Times.*

